# A genome-wide study of *de novo* deletions identifies a candidate locus for non-syndromic isolated cleft lip/palate risk

**DOI:** 10.1186/1471-2156-15-24

**Published:** 2014-02-14

**Authors:** Samuel G Younkin, Robert B Scharpf, Holger Schwender, Margaret M Parker, Alan F Scott, Mary L Marazita, Terri H Beaty, Ingo Ruczinski

**Affiliations:** 1 Department of Biostatistics, Johns Hopkins Bloomberg School of Public Health, Baltimore MD, USA; 2 Department of Oncology, Johns Hopkins School of Medicine, Baltimore MD, USA; 3 Mathematical Institute, Heinrich Heine University, Düsseldorf, Germany; 4 Department of Epidemiology, Johns Hopkins Bloomberg School of Public Health, Baltimore MD, USA; 5 McKusick-Nathans Institute of Genetic Medicine, Johns Hopkins School of Medicine, Baltimore MD, USA; 6 School of Dental Medicine, University of Pittsburgh, Pittsburgh PA, USA

**Keywords:** Oral clefts, DNA copy numbers, *de novo* deletions, Case-parent trios

## Abstract

**Background:**

Copy number variants (CNVs) may play an important part in the development of common birth defects such as oral clefts, and individual patients with multiple birth defects (including clefts) have been shown to carry small and large chromosomal deletions. In this paper we investigate *de novo* deletions defined as DNA segments missing in an oral cleft proband but present in both unaffected parents. We compare *de novo* deletion frequencies in children of European ancestry with an isolated, non-syndromic oral cleft to frequencies in children of European ancestry from randomly sampled trios.

**Results:**

We identified a genome-wide significant 62 kilo base (kb) non-coding region on chromosome 7p14.1 where *de novo* deletions occur more frequently among oral cleft cases than controls. We also observed wider *de novo* deletions among cleft lip and palate (CLP) cases than seen among cleft palate (CP) and cleft lip (CL) cases.

**Conclusions:**

This study presents a region where *de novo* deletions appear to be involved in the etiology of oral clefts, although the underlying biological mechanisms are still unknown. Larger *de novo* deletions are more likely to interfere with normal craniofacial development and may result in more severe clefts. Study protocol and sample DNA source can severely affect estimates of *de novo* deletion frequencies. Follow-up studies are needed to further validate these findings and to potentially identify additional structural variants underlying oral clefts.

## Background

Oral clefts are among the most common birth defects, and include three anatomical defects: cleft lip (CL), cleft lip and palate (CLP) and cleft palate (CP). Because there are similarities in embryological and epidemiological evidence [1,2], CL and CLP are often grouped together as cleft lip with/without cleft palate (CL/P), although debate remains about whether all three groups may have distinct etiologies [3,4]. Collectively, oral clefts represent the most common type of craniofacial malformations [5] and create a major public health burden for both affected children and their families. The overall birth prevalence of oral clefts is estimated at 1 per 700 live births worldwide, but there is dramatic variation across populations and between racial and ethnic groups, in particular for CL/P [2]. Oral clefts show strong familial aggregation, and the recurrence risk among first degree relatives is approximately 32 times greater than the general population risk for CL/P, and approximately 56 times greater for CP [6]. Twin studies also suggest a major role for genes controlling risk of oral clefts with monozygotic twins showing much higher concordance rates than dizygotic twins: 31% versus 2% for CL/P, and 43% versus 7% for CP [7]. Normal development of craniofacial features is a complex process and disruption of any of numerous steps can lead to development of oral clefts [8]. This etiologic complexity is further supported by mounting evidence that multiple genes or their regulatory genetic elements, in addition to environmental influences, play a role in the etiology of oral clefts, although supporting evidence for relatively few genes would be considered definitive [9-14].

Assessment of chromosomal anomalies such as microdeletions and translocations have played an important role in identifying genes and genomic regions underlying craniofacial disorders [15-23]. In particular, high throughput technologies such as comparative genomic hybridization (CGH) and single nucleotide polymorphism (SNP) arrays have gained popularity in identifying chromosomal alterations [24,25]. Sivertsen et al. assessed the prevalence of duplications and deletions in the 22q11 region (DiGeorge syndrome region) among Norwegian offspring with CP, but did not detect any association [26]. Shi et al. used SNP genotyping, DNA sequencing, high-resolution DNA microarray analysis, and long-range PCR to characterize chromosomal deletions in 333 candidate genes for orofacial clefting in 2,823 samples from 725 two and three generation families ascertained through a proband with a CL/P [27]. These authors confirmed several *de novo* deletions (defined as DNA segments missing in an oral cleft proband but present in both parents in two copies) in some of these candidate genes, in particular *SUMO1*, *TBX1*, and *TFAP2A*, raising the possibility that genes or regulatory elements contained within deleted regions might play a role in the etiology of oral clefts. Further, high rates of Mendelian inconsistencies were observed in 11 different genes, suggesting the existence of additional micro-deletions among oral cleft cases.

Family-based study designs as used by Shi et. al [27] are a popular alternative to the more common population-based designs (e.g. case-control studies) to assess associations between copy number variants (CNVs) and a disorder of interest [28-32], since investigating parents and offspring simultaneously enables the researcher to infer structural variants that occur *de novo* in the offspring (typically through a germline deletion). However, while numerous methods for CNV delineation in individual samples [33-38] or multiple independent samples [39-42] are available, only relatively few statistical approaches for detecting *de novo* CNVs have been proposed, and these are limited to offspring-parent trios. *PennCNV*[43] is based on a hidden Markov model (HMM), jointly modeling the unknown copy number states in all three trio members. Maximum likelihood methods are then employed to identify the most likely copy number states in the father, mother and offspring, which includes *de novo* deletions in the proband as a special case. *MinimumDistance*[44] on the other hand was specifically developed for detecting *de novo* deletions in case-parent trios, since the computational demands of the *PennCNV* joint HMM are substantial, and false positive calls of *de novo* deletions remain a concern even when the recommended quality control corrections are employed [44]. *MinimumDistance* captures differences in copy number estimates between the offspring and each parent at each locus before smoothing and posterior calling are carried out (see Methods), which greatly reduces technical and experimental sources of noise such as genomic waves, probe effects and batch effects [45,46], which are the major sources of false positive identifications in copy number analyses. Here, we employ both *MinimumDistance* and *PennCNV* to estimate and compare frequencies of *de novo* deletions in cleft probands and unaffected children from trios of European ancestry.

## Results

**Figure 1:**
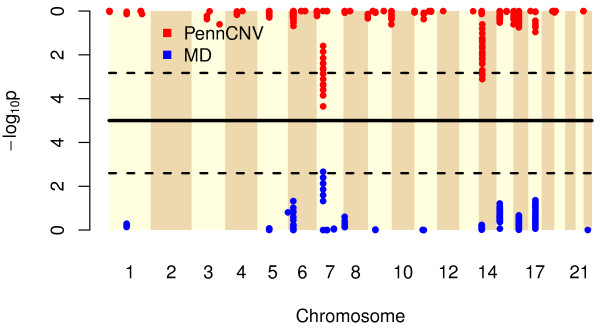
**The -log_10 _p-values (y-axis) derived from testing associations of inferred *de novo *deletions and oral clefts, shown by chromosomal location (x-axis).** Each point represents a *de novo* deletion CNV segment, delineated through *MinimumDistance*[44] (lower half) or *PennCNV*[43] (upper half). The dashed lines represent the genome-wide significance levels for a family-wise error rate of 5%, derived via permutation tests. The striped vertical bands indicate the 22 autosomes interrogated.

We compared the frequencies of *de novo* deletions in cleft probands and control children from trios. We identified *de novo* deletions in 467 cleft and 391 control trios, and found a 62 kilo base (kb) non-coding region on chromosome 7p14.1 where *de novo* deletions occurred significantly more often among the cleft trios. Two different algorithms were employed to delineate *de novo* deletions in the probands – *MinimumDistance*[44] and *PennCNV*[43] – and yielded a total of 190 and 455 CNV regions, respectively, where at least five *de novo* deletions occurred in both sets of trios combined. A significantly higher rate of *de novo* deletions in the cleft trios compared to control trios was observed near the 38.3 MB region on chromosome 7p14.1 (Figure 1; *p*=4.3×10^-2^ and *p*=1.1×10^-3^ respectively, corrected for multiple comparisons). This exact genomic region has been previously identified as a region with high structural variation (projects.tcag.ca/variation/), and deletions in this area have been associated with developmental problems including craniofacial abnormalities [47-51].

**Figure 2:**
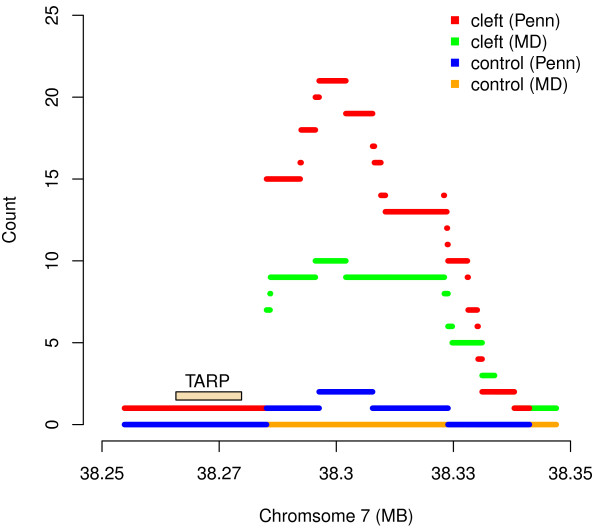
**The count of CNV components (y-axis) delineated via *MinimumDistance *in the cleft lip/palate trios (green) and control trios (yellow), as a function of genomic location (x-axis) near 7p14.1, plus the corresponding numbers delineated via *PennCNV *in the cleft lip/palate trios (red) and control trios (blue).** Segments are defined as collections of probes where CNV composition does not change in the combined trio sets. Short lines thus represent small differences in width among *de novo* deletions. The most significant association was observed in a sub-region where *MinimumDistance* (*PennCNV*) identified 10 (20) cleft lip/palate subjects with a *de novo* deletion, and none (one) in the control trios. The nearest gene is the T cell receptor gamma alternate reading frame protein *TARP*, about 20 kb away from the *de novo* deletions.

**Figure 3:**
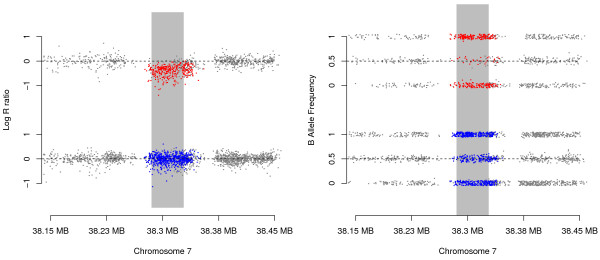
**The log R ratios (left) and B allele frequencies (right) near the identified 7p14.1 locus.** The upper panels (containing the red dots) represent the 10 oral cleft probands with *MinimumDistance* inferred *de novo* deletions at this locus, the lower panels (containing the blue dots) show the data for the parents of these probands. For each subject (parent or proband), color was used for the markers within the inferred *de novo* deletions (which differ in length between trios), gray dots were used for markers outside the deletions. The vertical gray bar indicates the segment of markers that yielded the most significant association test. For visualization, slight horizontal jitter was applied for both plots, and vertical jitter was applied for the B allele frequency plot.

The most significant association was observed in a sub-region where *MinimumDistance* (*PennCNV*) identified 10 (20) cleft cases with an apparent *de novo* deletion, and none (one) among the control trios (Figure 2). The 10 (20) case probands with *de novo* deletions in this region included 6 (9) CL, 3 (6) CP, and 1 (5) CLP cases. The nearest gene to this 7p14.1 region, about 20 kb upstream from the peak of this signal, is the T cell receptor gamma alternate reading frame protein (*TARP*). While this particular gene to our knowledge has not been previously associated with craniofacial abnormalities *per se*, copy number changes in T cell receptors (including those on 7p14) have been strongly associated with developmental problems [51]. For the 44 probes contained in this segment of 7p14.1, the signal intensities show a clear reduction among the 10 cases identified by *MinimumDistance*, indicating hemizygous deletions. These lower log R ratios were not observed in their parents, indicating a normal DNA copy number state (Figure 3). Sufficient DNA was available for three of the cleft trios with an inferred hemizygous *de novo* deletion at this region. Quantitative real-time PCR confirmed a clear copy number decrease in the child relative to his/her parents (Additional file 1). While *TARP* is not a very strong candidate for a causal gene per se, *HOXA2* on 7p14.2 is a functional candidate just over 1Mb away. A mutation in *HOXA2* causes microtia (deformity of the external ear), hearing impairment and cleft palate (http://www.omim.org/entry/604685). Though purely speculative, it might be possible that a copy number variant involving a distal enhancer might cause clefting similar to the way an enhancer 1 Mb from *SHH* (sonic hedgehog) produces preaxial polydactyly [52].

A second region of potential interest was identified by *PennCNV* on chromosome 14, however, upon manual inspection of signal intensities, this region appeared to be a false positive result (see Additional file 1). An analysis of *de novo* deletions called by both *MinimumDistance* and *PennCNV* also yielded the chromome 7p14.1 locus as the only significant finding among 90 CNV components from 11 distinct loci that had at least 5 de novo deletions called by both methods, with nine *de novo* deletions in cleft trios compared to none in the controls (*p*=0.032, corrected for multiple comparisons). It is also noteworthy that among the oral cleft candidate genes examined by Jugessur et al [11] and Shi et al [27], we only detected one inherited deletion (in *UGT1A7*), and no *de novo* deletions in these trios.

Another technique to infer or confirm *de novo* deletions, based solely on genotypes, is to search for clusters of Mendelian inconsistencies between genotypes of the trio [27,53]. In our study however, the identified regions on chromosome 7 were small and the corresponding SNPs interrogated by these probes had low minor allele frequency in our population, so no Mendelian inconsistencies were observed among the trios with an inferred *de novo* deletion in the proband.

Comparing the overall widths of *MinimumDistance* and *PennCNV* inferred *de novo* deletions in cleft cases and controls revealed that the estimated deletions were substantially larger in cases than in controls. The median deletion width inferred from *MinimumDistance* (*PennCNV*) was 71.7 kb (61.3 kb) among controls and 102.7 kb (70.5 kb) among cases, corresponding to an increase of 43% (15%) in median width of a deletion among cases (Table 1). These observed differences in widths were statistically significant (Kolmogorov-Smirnov p-values of 1.2×10^-4^ and 2.9×10^-3^ respectively, Wilcoxon rank-sum p-values of 1.0×10^-4^ and 5×10^-2^ respectively; see Methods). Compared to the controls, the *MinimumDistance* inferred *de novo* deletions were also larger when cleft types were considered individually, increasing from CL (median 87.8 kb) to CP (95.2 kb) to CLP (128.5 kb). For inferred *de novo* deletions identified by *PennCNV*, we did not observe any trend of increasing size by cleft type, as CP deletions (median 52.5 kb) were smaller than apparent deletions in controls (Table 1). However, this observation may reflect an excess number of false positive (and mostly short) *PennCNV* identifications among controls, as discussed in more detail below.

**Table 1:** **The median width in kilo bases (kb) of *de novo *** **deletions, stratified by case status and cleft type for both discovery methods**

	**Controls**	**Clefts**	**p**	**CL**	**p**	**CP**	**p**	**CLP**	**p**
*MD*	71.7	102.7	1e-4	87.8	0.084	95.2	0.067	128.5	1e-5
*PennCNV*	61.3	70.5	0.051	81.1	0.006	52.5	0.923	85.7	0.041

*MD*: *de novo* deletions with coverage of at least ten markers inferred by *MinimumDistance*[44]; *PennCNV*: *de novo* deletions with coverage of at least ten markers inferred by *PennCNV*[43]. CL: cleft lip; CP: cleft palate; CLP: cleft lip and palate. The p-values were derived using a one-sided Wilcoxon rank-sum test to assess a potential increase in width of *de novo* deletions in the cleft offspring versus controls.

## Discussion

**Figure 4:**
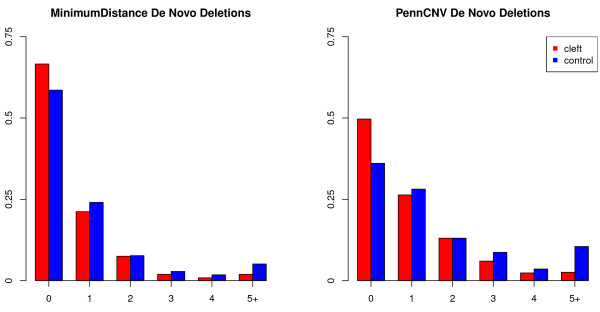
**The frequency of inferred *de novo *deletions per subject, inferred via *MinimumDistance *(left) and *PennCNV *(right), shown separately for oral cleft probands (red) and controls (blue).** Subjects with five or more called *de novo* deletions were grouped together.

Even though all trios with at least one sample of poor data quality were excluded (see Methods), the probe intensity signal used to identify regions of copy number changes was somewhat noisy, and substantially more variable among control trios than in the oral cleft trios, resulting in an inflated rate of called *de novo* deletions (i. e. likely false positives) in the control group (Figure 4). This effect was much more prominent in the set of *de novo* deletions identified by *PennCNV*, consistent with a previous observation that *MinimumDistance* might be more robust to false positive identifications (see Figure 2 in [44]). When delineated via *PennCNV*, the control group had more than a three-fold *de novo* deletion rate, and less than a two-fold rate when *de novo* deletions were inferred with *MinimumDistance* (Table 2). However, our statistical procedure for inferring *de novo* deletions employed in this study guards against spurious associations, and thus type I error inflation, due to higher rates of false *de novo* deletions called in the control trios, since we performed a one-sided test with the alternative hypothesis that the *de novo* deletion rate was larger among the cases than controls. In contrast, a two-sided test would not protect against this type I error inflation due to excessive false positives among the controls (see Additional file 1). We also note a one-sided hypothesis test would not guard against type I error inflation if higher variability in the control group would mask deletions. Thus, all significant findings should be carefully inspected, and validated if possible.

**Table 2:** **The total number of *de novo *** **deletions inferred (“count”) and the average number of inferred *de novo *** **deletions per child (“average”), stratified by case status and cleft type for both discovery methods**

		**Controls**		**Oral clefts**		**CL**		**CP**		**CLP**
*MD*	438	1.12	286	0.61	89	0.64	80	0.62	117	0.59
*PennCNV*	1,422	3.64	518	1.11	185	1.32	144	1.12	189	0.95
	count	average	count	average	count	average	count	average	count	average

*MD*: *de novo* deletions with coverage of at least ten markers inferred by *MinimumDistance*; *PennCNV*: *de novo* deletions with coverage of at least ten markers inferred by *PennCNV*. CL: cleft lip; CP: cleft palate; CLP: cleft lip and palate. All differences between cleft and control counts are highly significant, assuming independence of deletions (*p*<10^-6^ for all *MD* comparisons, *p*<10^-40^ for all *PennCNV* comparisons).

As DNA source is correlated with sample quality and affects all CNV call rates, we assessed and found substantial differences in proportions of DNA sources between cases and controls. Around 36% of the control samples were collected either by buccal swab, mouthwash or saliva, while only 17% of the cleft cases were extracted this way (Table 3). Among samples passing quality control (see Methods for details), the rate of inferred *de novo* deletions was much higher among samples where DNA was extracted from anything other than whole blood (see Additional file 1). We conjecture that the increased rate of called *de novo* deletions in the control group is likely driven by the differences in the DNA sample collections, with *MinimumDistance* being more robust to this artifact than *PennCNV*. Thus, for this particular study, the *MinimumDistance* based statistics and comparisons should be more reliable. We also note that false identifications tend to involve very short segments of DNA, based on fewer markers from the array. In short, false positive identifications can skew the distribution of CNV lengths, therefore we report the median deletion widths here.

**Table 3:** The total number (“count”) and relative frequencies (“proportion”) of DNA source types among all subjects, grouped by proband cleft/control status

		**Controls**		**Oral clefts**
blood	581	0.644	1139	0.828
buccal	64	0.071	125	0.091
mouthwash	50	0.055	21	0.015
saliva	207	0.229	90	0.065
	count	proportion	count	proportion

DNA source types differ significantly between cases and controls (*p*<10^-35^).

## Conclusions

We identified a genome-wide significant 62 kb non-coding region on chromosome 7p14.1 where *de novo* deletions occurred more frequently in oral cleft cases than control probands, adding to the evidence that structural variants are involved in the etiology of oral clefts. This region has been previously identified as a genomic region containing high structural variation, and large deletions in this region have been reported to result in developmental problems including craniofacial abnormalities [47-51]. Only 20 kb upstream from the signal peak lies the gene coding for the T cell receptor gamma alternate reading frame protein (*TARP*), adding to the existing literature that T cell receptors can play a role in human development. We also observed an overall increase in the width of *de novo* deletions among oral cleft probands, with CLP exhibiting wider *de novo* deletions than CL and CP cases. Study protocol and sample DNA source affect estimated frequencies of *de novo* deletions, and the problem of false positive identifications remains a concern when examining the role of structural variants from genomic array data.

## Methods

### Samples

Case-parent trios were collected as part of an international collaborative study in the GENEVA Consortium [54]. These trios were ascertained through probands with an isolated, non-syndromic oral cleft (either cleft lip, cleft palate or cleft lip and palate) from 13 different recruitment sites in the United States, Europe, Southeast and East Asia [12]. Control trios were derived from small pedigrees collected from rural Appalachia as part of a genome-wide study of dental caries [55]. The DNA sources for cleft trio samples included whole blood, buccal brush/swab, saliva, mouthwash and dried blood spots, and varied by recruitment site. DNA sources for control trios also included whole blood, buccal brush/swab, saliva, and mouthwash. All samples were hybridized to the Illumina Human610-Quad Beadchip and typed at the Center for Inherited Disease Research (CIDR) at Johns Hopkins University (http://www.cidr.jhmi.edu/). This research project complies with the Helsinki Declaration and all participating institutions provided their own institutional review board (IRB) review and approval, in addition to the review and approval of the Johns Hopkins School of Public Health IRB for the collaborative analysis of genome-wide marker data. Written informed consent was obtained from parents of children ascertained through an oral cleft, as well as their own consent or assent when the proband could appropriately give such. To avoid potential confounding due to ethnic differences (i.e. genetic background), we restricted our analysis to subjects of European ancestry only.

Both *MinimumDistance* and *PennCNV* utilize the log R ratios (LRRs) and B allele frequencies (BAFs) from the Illumina Human610-Quad Beadchip probes to infer *de novo* deletions. The LRR is a standardized estimate of the probe intensity, quantifying the total number of allele copies at each locus of interest. The BAF is a standardized estimate for the proportion of the B allele’s contribution to the total probe intensity, assessing the genotype at the locus of interest. The BAF is standardized so homozygous genotypes in copy neutral states (two allele copies) have BAFs of approximately zero or one (for AA and BB genotypes, respectively), and heterozygous AB genotypes yield BAFs roughly equal to 0.5. Following previously established guidelines for quality control [43,44] devised particularly to avoid excessive false positive identifications due to poor data quality, we excluded trios for which any sample had whole genome amplified DNA or a LRR median absolute deviation (MAD) above 0.3. We also excluded trios with members flagged by CIDR’s internal quality control pipeline. These data cleaning procedures yielded 467 oral cleft trios composed of 1,375 subjects, and 391 trios composed of 902 subjects as controls. Aside from the CNV discovery via *PennCNV*, all analyses were carried out in the statistical environment R (http://cran.r-project.org/) using the packages *DNACopy, GenomicRanges, GWASTools, IRanges, MinimumDistance*, all available as free software via the Bioconductor project (http://www.bioconductor.org/) [56].

### Algorithms

The *PennCNV* algorithm for detection of *de novo* DNA copy number aberrations is based on a hidden Markov model (HMM), jointly modeling the (unknown) copy number states in all three trio members. The state transition probabilities are based on the observed LRRs and BAFs in the samples, and the population BAF. Maximum likelihood methods are employed to identify the most likely copy number states in the father, mother and offspring, and these are encoded as a three-digit numerical code. A normal DNA copy number (two alleles) is designated as a 3, a hemizygous deletion (one allele copy) is indicated as a 2, and a homozygous deletion (zero allele copies) is indicated as a 1. Thus, *de novo* deletions in offspring with genotypic normal parents are encoded as trio state ‘332’ (loss of one allele copy in the child) or ‘331’ (loss of both alleles). *PennCNV* addresses genomic waves by incorporating the population GC content at each marker into the HMM.

While the joint *PennCNV* HMM considers all possible copy number states including inherited deletions (e.g. ‘322’ or ’232’), *MinimumDistance* was developed specifically for detecting *de novo* copy number changes since the computational demands of the joint *PennCNV* HMM are substantial, and false positive identifications of *de novo* deletions remain a concern even when the recommended quality control procedures (including genomic wave correction) are employed [44]. This approach, freely available as a Bioconductor package (http://www.bioconductor.org/), is based on the “minimum distance” statistic, capturing differences in copy number estimates between the offspring and each parent at each locus, making it robust to genomic waves by design. In particular when the samples of the trio members are hybridized on the same plate (which is the highly recommended and commonly employed approach), *MinimumDistance* is an effective approach for reducing technical and experimental sources of noise which can generate false positives in experimental data sets. Following genome-wide segmentation of these minimum distances by circular binary segmentation [34,57] (an extremely fast procedure), final inference regarding *de novo* copy number events is based on a posterior calling step on the inferred candidate regions. *MinimumDistance* uses the same code for the trio copy number states as *PennCNV*, where ‘332’ and ‘331’ represent *de novo* loss of alleles in the child.

### Inference

To test for association with oral cleft status, we compared CNV components among cases and controls. Since inferred deletions (required to span at least 10 probes on the array for our analysis) typically only partially overlap between trios, we used the *IRanges* package to delineate the CNV components into sets of markers where no change in copy number state occurred among any of the cleft or control trios, defining homogeneous sets of CNV states (see Additional file 1). For all CNV components with a total of at least five observed *de novo* deletions in the cleft and control trios combined, we performed a one-sided Fisher’s exact test, where the alternative hypothesis was a higher *de novo* deletion frequency in the cleft probands. To correct for multiple comparisons while simultaneously taking correlations between component statistics into account, we performed a permutation test by shuffling case and control status across all probands. This procedure, based on over 100,000 permutations, established the genome-wide significance level for a 5% family-wise error rate at the nominal values of 2.60 and 2.83 for the – log10 p-values for *MinimumDistance* and *PennCNV*, respectively. We also performed simulations to compare the widths of *de novo* deletions in cleft and control trios. More specifically, we simulated 10,000 quantile-quantile plots under the assumption that the cleft and control samples came from the same distribution (see Additional file 1), and used a one sided two-sample Kolmogorov-Smirnov test to assess a potential increase in width of *de novo* deletions in the cleft offspring. Since non-parametric mean comparisons might be less sensitive to subtle batch effects on deletion width, we also carried out a one-sided Wilcoxon rank-sum test on the observed deletion widths in the case and control trios.

## Competing interests

The authors declare no competing interests.

## Authors’ contributions

SGY, RBS, HS and MMP wrote all code, performed all analyses, and generated all tables and figures. THB and IR conceived of the study. All authors participated in its design, coordination, and drafting of the manuscript. All authors read and approved the final version of the manuscript.

## Supplementary Material

Additional file 1 Supplementary material. Click here for file
