# Supplementary material for: A genome-wide study of de novo deletions identifies a candidate locus for non-syndromic isolated cleft lip/palate risk
Source: BMC Genet. 2014 Feb 14;15:24. doi: 10.1186/1471-2156-15-24 (PMC3929298; doi:10.1186/1471-2156-15-24)
Supplement: Additional file 1 — Supplementary material. [file 1471-2156-15-24-S1.pdf]

# A genome-wide study of de novo deletions identifies a candidate locus for non-syndromic isolated cleft lip/palate risk

# Supplementary Material

Samuel G Younkin*[1], Robert B Scharpf[2], Holger Schwender[3], Margaret M Parker[4], Alan F Scott[5], Mary L Marazita[6], Terri H Beaty[4] and Ingo Ruczinski[1]

[1] Department of Biostatistics, Johns Hopkins Bloomberg School of Public Health, Baltimore, MD, USA; [2] Department of Oncology, Johns Hopkins School of Medicine, Baltimore, MD, USA; [3] Mathematical Institute, Heinrich-Heine-University, Düsseldorf, Germany; [4] Department of Epidemiology, Johns Hopkins Bloomberg School of Public Health, Baltimore, MD, USA [5] McKusick-Nathans Institute of Genomic Medicine, Johns Hopkins School of Medicine, Baltimore, MD, USA; [6] School of Dental Medicine, University of Pittsburgh, Pittsburgh, PA, USA;

Email: syounkin@jhsph.edu;

*Corresponding author

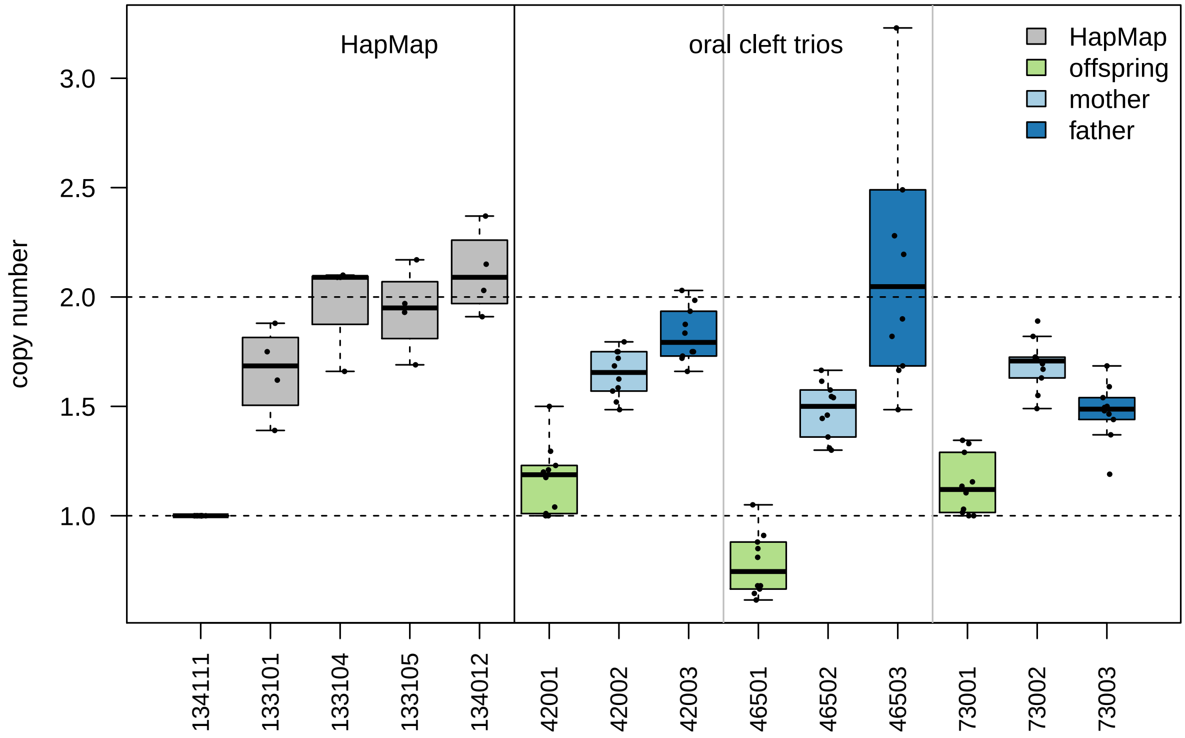


Figure S1: We used quantitative real-time PCR to validate 3 apparent *de novo* hemizygous deletions inferred from the Illumina arrays. Boxplots are used to summarize the copy number estimates (y-axis) obtained from 10 TaqMan probes located within the putative *de novo* deletion site for 14 samples (a boxplot for each sample). The samples include a positive control with an apparent hemizygous deletion (far left), 3 HapMap negative controls presumed to be diploid (boxplots 2-5), and 3 case-parent trios. The copy number estimate for each TaqMan probe was obtained using the CopyCaller software (v2.0) provided by the manufacturer with sample 134111 (HapMap id NA07034, far left) to calibrate hemizygous deletions.

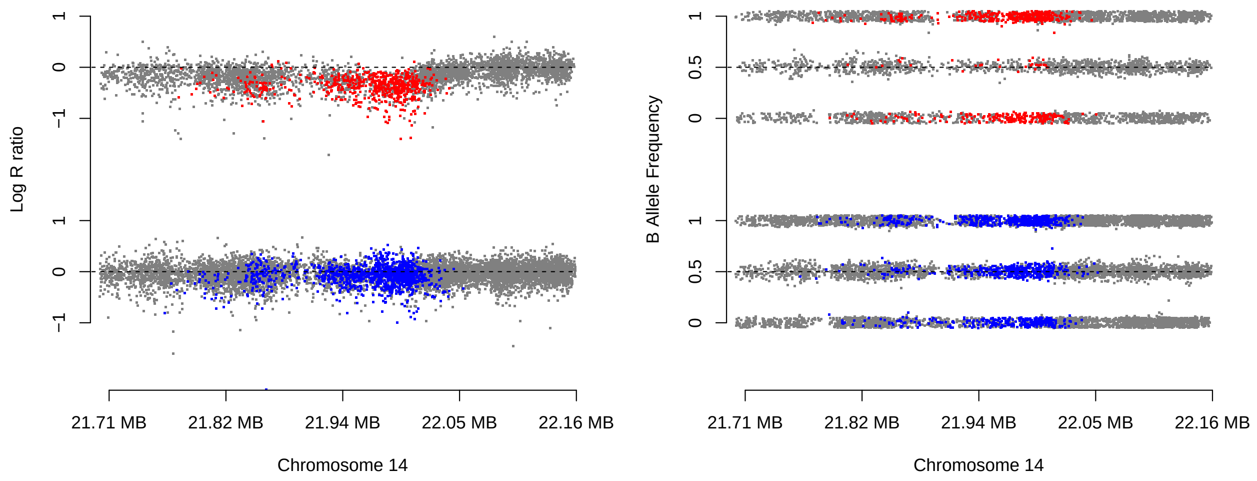


Figure S2: The log R ratios (left) and B allele frequencies (right) near the *PennCNV* finding on chromsome 14. This region contains three sub-regions (separated by 47.7 kB and 6.9 kB) with "testable" components, that is contiguous sets of loci where at least five *de novo* deletions were called by PennCNV in cleft and control trios combined. The first two regions have one testable component each, with five PennCNV inferred de novo deletions in the cleft trios, and none in the control trios. The third region is 90.8 kb wide, and consists of 24 components. The most significant component among those has 15 *de novo* deletions inferred in the cleft trios, and 1 in the control trios ($p = 0.0008$). The component with the most deletions has 19 *de novo* inferred deletions in the cleft trios, and 4 in the control trios. The upper panels (containing the red dots) represent the total of 23 oral cleft probands with *PennCNV* inferred *de novo* deletions in either of these regions, the lower panels (containing the blue dots) show the data for the parents of these probands. For each subject (parent or proband), color was used for the markers within the inferred *de novo* deletions (which differ in length between trios), grey dots were used for markers outside the deletions. For visualization, slight horizontal jitter was applied for both plots, and vertical jitter was applied for the B allele frequency plot. A large "genomic wave" is visible, with reduced log R ratios for the probands in most of the region.

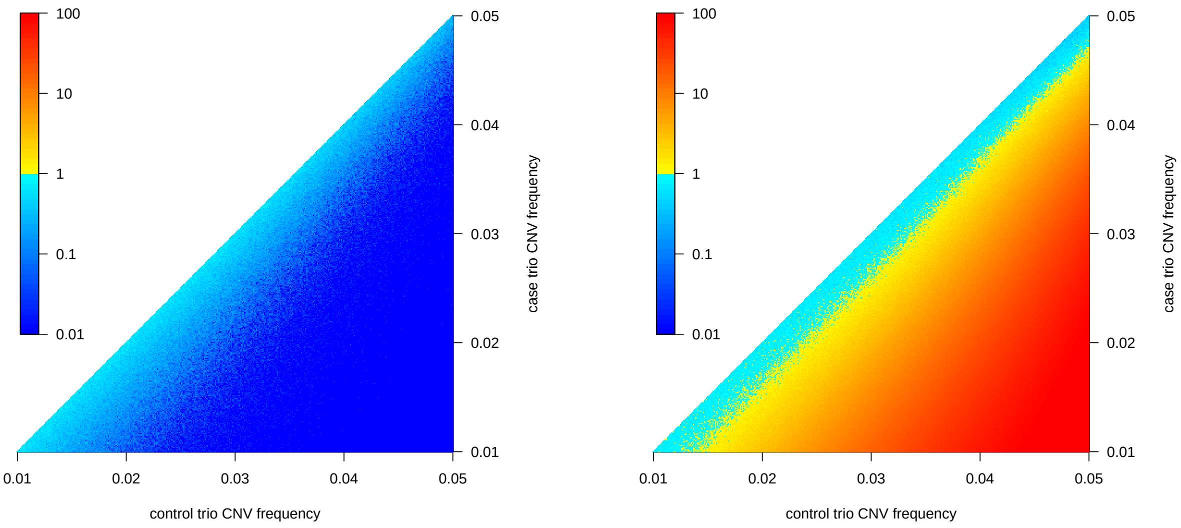


Figure S3: Type I error inflation in the presence of false positive CNV identifications for one-sided (left) and two-sided (right) hypothesis tests. The simulation was based on the actual data assuming 467 case trios, 391 control trios, and a critical value of 2.60 for statistical significance (corresponding to a p-value of 0.0047). We assumed values between 1% and 5% for *called* CNV frequencies in case and control trios (in regions without any *de novo* deletions, these frequencies therefore rerpresent expected fractions of false positive identifications). Since control trios are noisier, we assumed that control trio frequencies of called *de novo* deletions can never be smaller than case trio frequencies. For each case and control trio frequency combination (simulated on a 401 by 401 grid without smoothing) we ran 10,000 iterations, taking draws from two independent Binomial distributions with the respective numbers of trios and frequencies to simulate observed numbers of *de novo* deletions and executing Fisher's exact test (one- and two-sided) for each instance. The respective colors in the image represent the ratio of the proportion of significant hypothesis tests ($p \leq 0.0047$) over the significance level ($p = 0.0047$), truncated at 100 and 1/100, respectively. Thus, red colors represent type I error inflation above the targeted significance level, and blue colors the opposite. The one-sided test (with the alternative hypothesis that the de novo deletion rate is larger among case than control trios) guards against spurious associations and thus type I error inflation due to higher rates of false de novo deletions called in the control trios (left), while in contrast, the two-sided test does not generally protect against this type I error inflation due to excessive false positives among the controls.

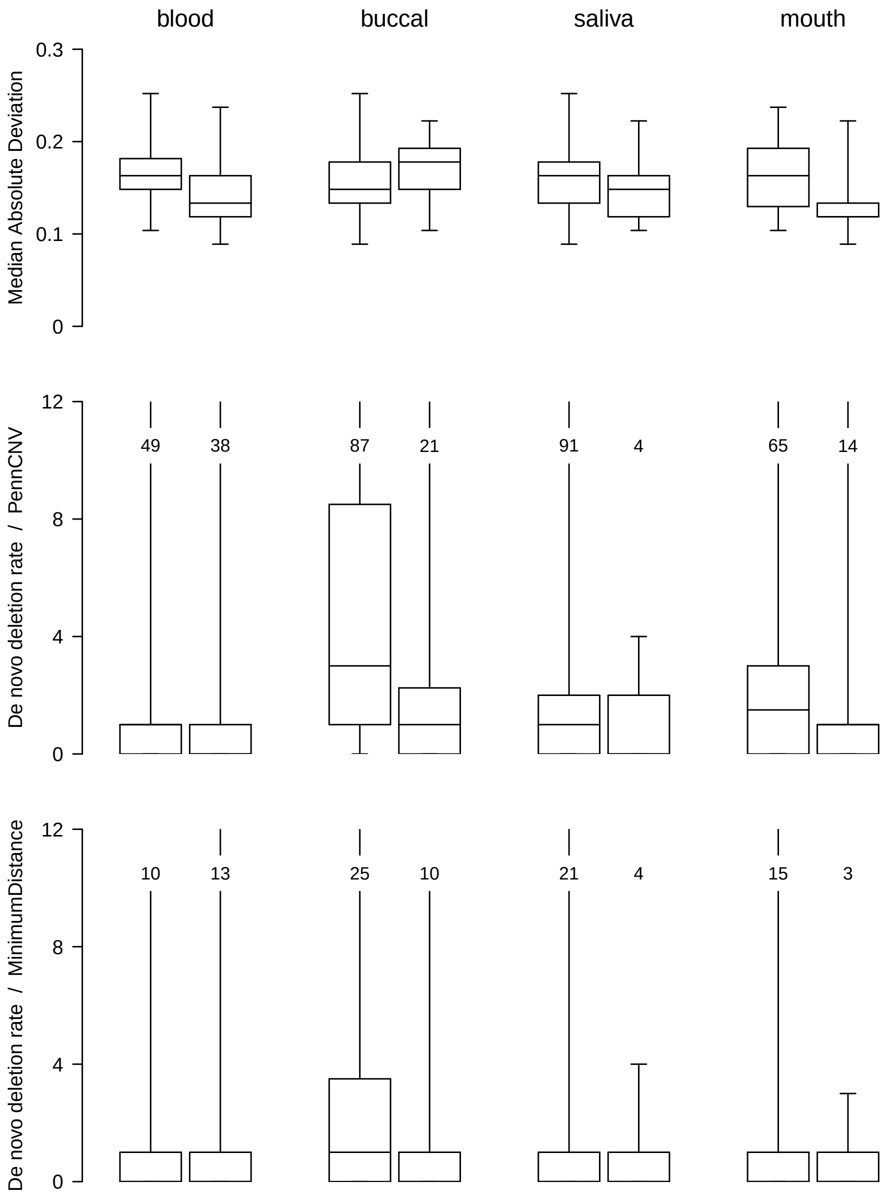


Figure S4: Summaries of the median absolute deviation of autosomal log R ratios (upper row) and the *de novo* deletion call rates (calls per sample; *PennCNV* middle row, *MinimumDistance* lower row) by DNA source (blood, buccal, saliva, mouth). The pair of boxplots in each panel represent the distribution in the control (left) and oral cleft trios (right). The whiskers extend to the minimum and maximum observed values, but were truncated at 12 in the *de novo* deletion rate panels for clarity. The numbers in these panels indicate the actual maximum value for the respective group.

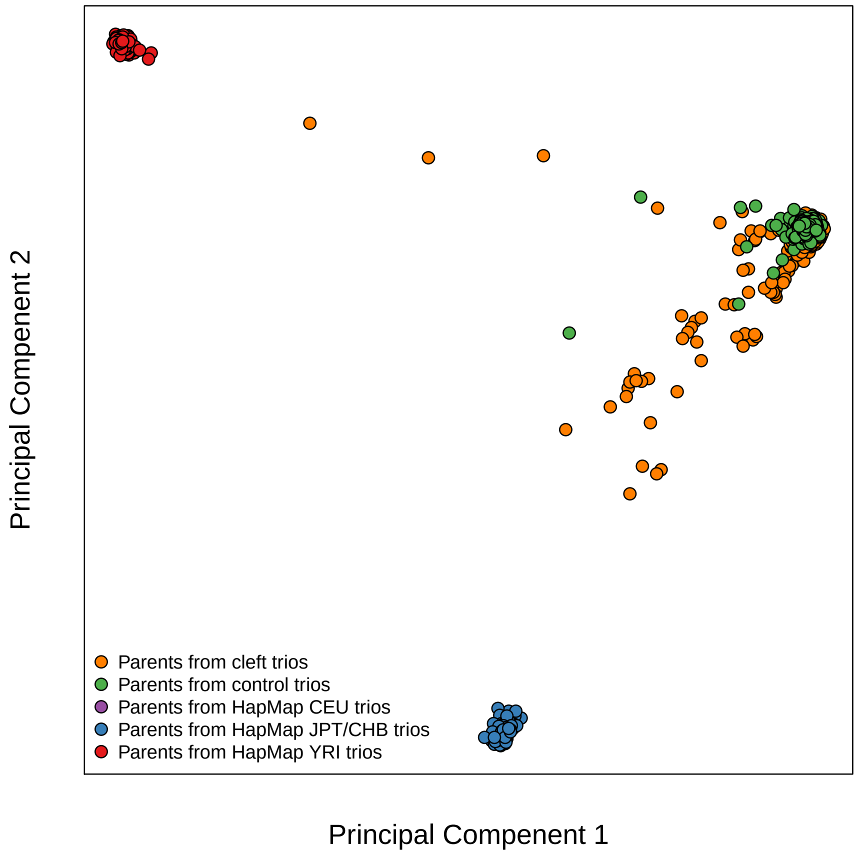


Figure S5: Genetic background of cleft and control trios displayed by the results of a principal components analysis. Both cleft and control trios are of European ancestry, with a modest amount of admixture observed in some samples.

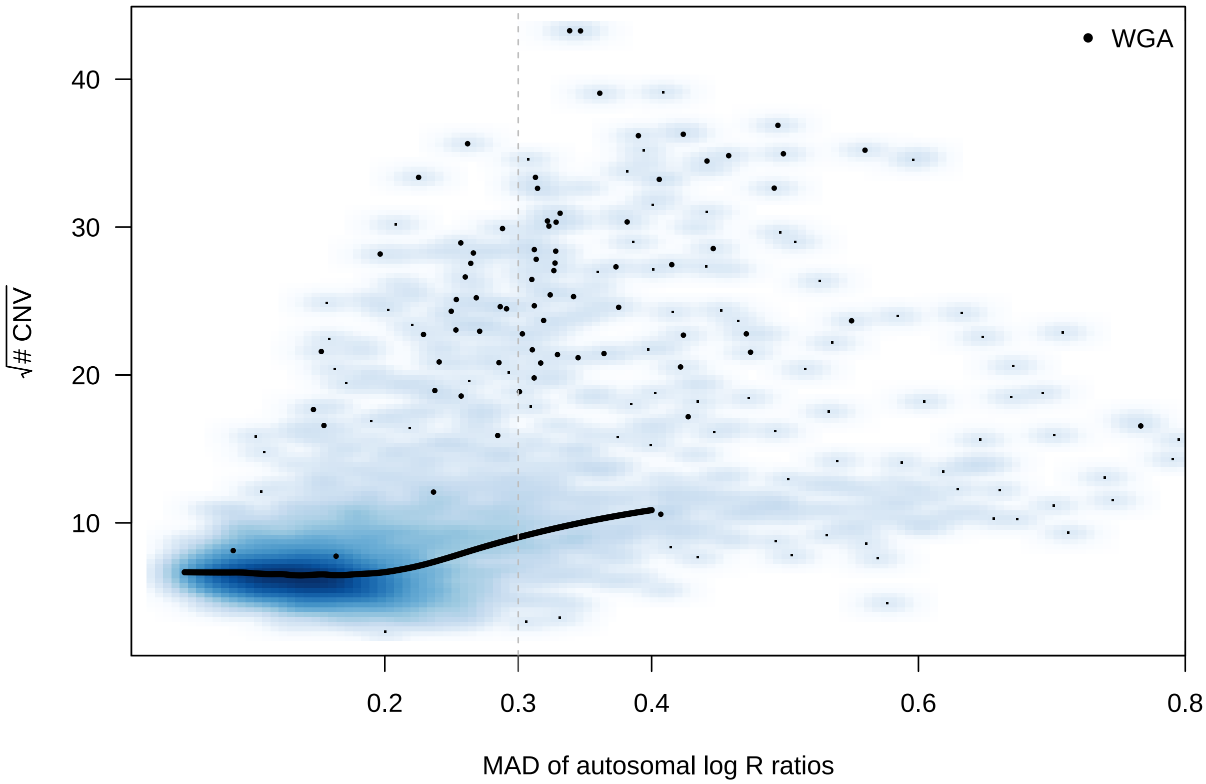


Figure S6: The median absolute deviation of the autosomal log R ratios (x-axis) versus the number of PennCNV inferred CNVs (y-axis, square root transformed) in the samples of the cleft and control trios combined. The loess curve was fit excluding whole genome amplified samples (indicated by larger circles), and samples with extreme call frequencies (greater than 15 on this scale, i.e. 225 in actual counts). Larger variability in the log R ratios translates into higher CNV call rates.

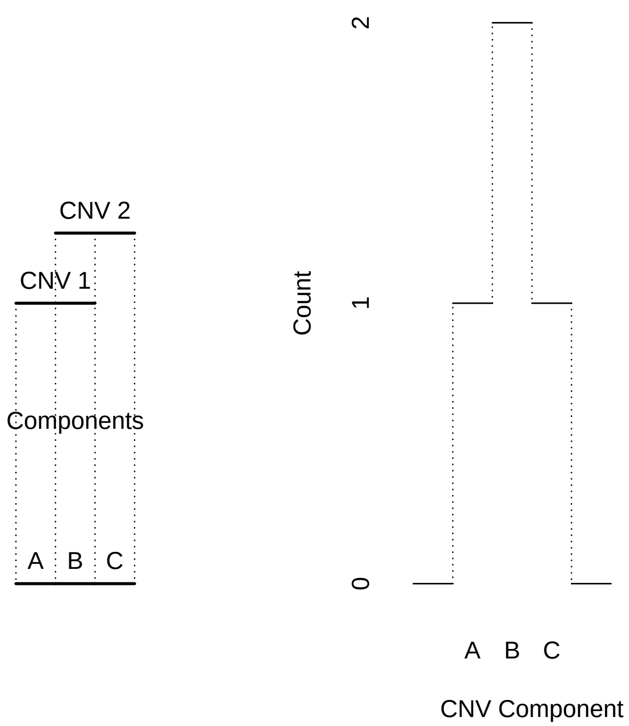


Figure S7: CNV components are constructed by decomposing the set of all inferred *de novo* deletions into sets of loci where no change of copy number state occurrs among any of the oral cleft or control trios, defining homogeneous sets of CNV states. In this example two partially overlapping CNVs (left) are decomposed into three CNV components (right) containing one (A), two (B) and one (C) trio with a *de novo* deletion. The CNV components are the units tested for association with oral cleft.

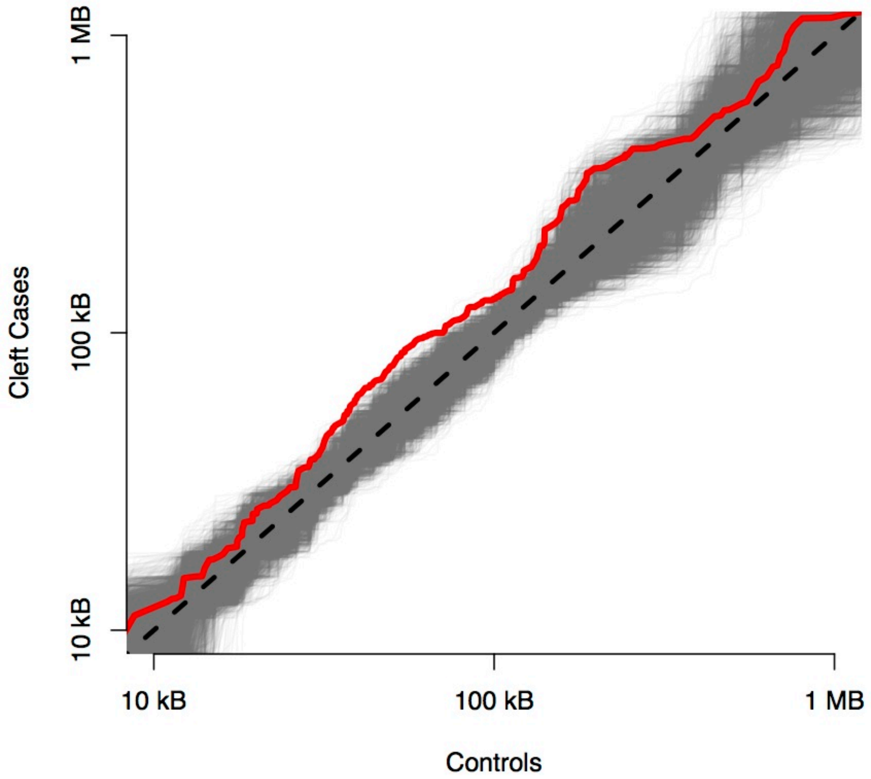


Figure S8: Quantile-quantile plot comparing the widths of the inferred *de novo* deletions in control (x-axis) and oral cleft trios (y-axis). Ten thousand samples from an empirical null distribution for both the cleft and control groups provided the data for the quantile-quantile plots in gray. The empirical null distribution was computed under the assumption that the cleft and control samples came from the same distribution, and empirical quantiles were found at each of the one-tenth percentiles for the combined cleft and control data. We sampled with replacement from the vector of quantiles to simulate draws from the null distribution. In this example, the *de novo* deletions were inferred with *MinimumDistance*.
